# Bacterial endophthalmitis following anti-VEGF intravitreal injections: a retrospective case series

**DOI:** 10.1186/s40942-023-00490-9

**Published:** 2023-09-26

**Authors:** Vinicius Campos Bergamo, Luis Filipe Nakayama, Nilva Simeren Bueno De Moraes, Maria Cecília Zorat Yu, Ana Luiza Höfling-Lima, Maurício Maia

**Affiliations:** 1 Retina Division, Department of Ophthalmology, Escola Paulista de Medicina, Federal University of São Paulo, 806, Botucatu Street, São Paulo, 04026-062 Brazil; 2 Laboratory of Ocular Microbiology, Escola Paulista de Medicina, Federal University of São Paulo, São Paulo, Brazil; 3 Cornea and External Diseases Division, Escola Paulista de Medicina, Federal University of São Paulo, São Paulo, Brazil; 4 Laboratory for Computational Physiology, Institute for Medical Engineering and Science, Massachusetts Institute of Technology, Cambridge, MA USA

**Keywords:** Anti-VEGF, Endophthalmitis, Intravitreal injections, Retina, Vitrectomy

## Abstract

**Background:**

To describe the incidence of endophthalmitis and the treatment outcomes of acute bacterial endophthalmitis following intravitreal anti-vascular endothelial growth factor (anti-VEGF) injections in a Brazilian hospital. The analysis was based on the timing of infection after intravitreal injection, culture results, visual acuity, and the presence of epiretinal membrane after a 1-year follow-up period, spanning nine years.

**Methods:**

This retrospective case series, conducted over a 9-year period, aimed to evaluate the treatment outcomes of acute endophthalmitis following intravitreal Bevacizumab injections. The inclusion criteria involved a chart review of 25 patients who presented clinical signs of acute endophthalmitis out of a total of 12,441 injections administered between January 2011 and December 2019. Negative culture results of vitreous samples or incomplete data were excluded. Ultimately, 23 patients were enrolled in the study. Eight patients were treated with intravitreal antibiotic injections (IVAI) using vancomycin 1.0 mg/0.05mL and ceftazidime 2.25 mg/0.05mL, while 15 patients underwent pars plana vitrectomy (PPV) followed by intravitreal antibiotic injections at the end of surgery (IVAIES). The main outcome measures were the efficacy of controlling the infection with IVAI as a standalone therapy compared to early PPV followed by IVAIES. Data collected included pre-infection and one-year post-treatment best corrected visual acuity (BCVA), optical coherence tomography (OCT) abnormalities, and enucleation/evisceration rates. To compare groups, Mann-Whitney and ANOVA tests were employed for statistical analysis.

**Results:**

The incidence rate of bacterial endophthalmitis was 0.185% (1/541 anti-VEGF injections), with the highest infection rates observed in 2014 and 2017. Patients presented clinical symptoms between 2 and 7 days after injection. The most common isolated organisms were coagulase-negative *Staphylococci* and *Streptococci spp*. Treatment outcomes showed that both IVAI and PPV + IVAIES effectively controlled the infection and prevented globe atrophy. After one year, the PPV group with BCVA better than Light Perception had a significantly better BCVA compared to the IVAI group (p 0.003). However, PPV group had higher incidence of epiretinal membranes formation compared to the IVAI group. (P 0.035)

**Conclusion:**

Anti-VEGF injections carry a risk of developing acute bacterial endophthalmitis. Isolated antibiotic therapy could be an effective treatment to control the infection, but performing PPV + IVAIES as a primary treatment showed promising results in terms of improving BCVA after one year, despite a higher rate of epiretinal membrane formation. Further studies are needed to confirm these findings.

## Introduction

Endophthalmitis is a rare but severe form of ocular inflammation secondary to an intraocular cavity infection that can lead to irreversible visual acuity (VA) loss if not treated appropriately in a timely manner [1]. Endophthalmitis can be classified based on time of infection presentation (acute or chronic), etiology (bacterial or fungal), transmission route (endogenous or exogenous), and organisms [2].

The Endophthalmitis Vitrectomy Study (EVS) was a multicentric, randomized clinical trial that established the basis for acute endophthalmitis management. The treatment consists of pars plana vitrectomy (PPV) in patients with light perception (LP) VA and intravitreal antibiotic therapy (IVAI) in patients with VA better than LP [3]. However, the EVS is almost three decades old and has limited treatment and diagnosis options. Recent studies have reported the efficacy of early IVAI with later PPV as an alternative therapy, considering the improvements in surgical equipment and technique, resulting in better outcomes [4].

Anti-vascular endothelial growth factor (anti-VEGF) intravitreal injections are the treatment of choice for retinal diseases such as diabetic macular edema (DME), retinal venous occlusions, and age-related macular degeneration (AMD); millions of injections are administered annually, and due to the repeated injection guidelines, the risk of developing acute endophthalmitis could be increased [5].

To date, no clinical trial has addressed the treatment for acute endophthalmitis after intravitreal injections [6], and the EVS guidelines supports treatment for acute endophthalmitis regardless of etiology [7–9].

We conducted a study on endophthalmitis cases that occurred after anti-VEGF injections at a Brazilian tertiary university hospital. The study included data over a 9-year period, and we compared patient demographics, ophthalmologic variables, and anatomic outcomes associated with different type of treatments.

## Methods

A single-center retrospective consecutive case series was conducted on patients from the Vitreoretinal Unit of Ophthalmology Department of Paulista School of Medicine, Federal University of São Paulo (UNIFESP), Brazil. The university’s Ethics Committee approved the study (number 0060/2018), and informed consent was waived due to its retrospective nature.

At the UNIFESP retinal unit, the most used anti-VEGF is bevacizumab (Avastin, Genentech, Inc., South San Francisco, CA, USA) and the treatment protocol is based on ophthalmologic and optical coherence tomography (OCT) findings.

For each suspected case, a vitreous sample was collected, and the microbiologic analysis was performed at the Ocular Microbiology Laboratory of the Department of Ophthalmology, Federal University of Sao Paulo. Vitreous culture was the sole diagnostic approach utilized for identifying endophthalmitis in these cases.

All patients were followed up with regular ophthalmologic and OCT examinations for a minimum of 1 year after the diagnosis of endophthalmitis.

### Study patients

The study encompassed data from all patients who received intravitreal anti-VEGF injections between January 2011 and December 2019. Presumed cases of endophthalmitis were identified by retrieving records from the UNIFESP Ocular Microbiology Laboratory. The eligibility of each suspected case was evaluated based on their clinical and laboratory history, including a history of ocular hyperemia, pain, as well as inflammation in the anterior chamber and vitreous. Only cases of microbiologically confirmed endophthalmitis were included in the analysis, while presumed or unconfirmed diagnoses were excluded.

### Variables analyzed

The analysis involves various aspects, including patient demographic characteristics, treatment types, initial best-corrected visual acuity (BCVA), BCVA immediately prior to treatment, BCVA one year after treatment, and anatomic parameters assessed through optical coherence tomography (OCT).

For the comparative analysis, the patients were divided into two groups based on their primary treatment approach. One group underwent PPV followed by an intravitreal antibiotic injection at the end of the surgery (IVAIES) (referred to as the PPV Group), while the other group received only an IVAI (referred to as the Injection Group). Within the PPV group, further divisions were made based on BCVA immediately prior to the surgery, categorized according to the study conducted by the Endophthalmitis Vitrectomy Study (EVS) [7]. The two subgroups created were the “worse-vision PPV group” (BCVA equal or worse than LP) and the “better-vision PPV group” (BCVA better than LP).

This division allowed for a comparative analysis between the different treatment groups based on their BCVA outcomes and the classification established by the EVS study.

### Surgical intervention

In the first group of patients, PPV was performed using 23-gauge instruments. A conservative approach was adopted based on visualization during the surgery (with an attempt to detach the posterior hyaloid only when it was possible and a conservative vitreous base shaving). Vitreous specimens were collected at the beginning of the surgery, and a balanced saline solution was used as a vitreous substitute. Sclerotomies were sutured with Vycryl 7.0 sutures. At the end of the surgery, intravitreal injection of 0.05 mL of vancomycin 1.0 mg and 0.05 mL of ceftazidime 2.25 mg was administered. During follow-up, all patients received topical moxifloxacin 0.3% treatment for 7 days, and the use of prednisolone acetate 1% was gradually tapered. All patients in this group were pseudophakic and had no reported previous complications. No oral antibiotics were prescribed.

In the second group of patients, treatment involved only an intravitreal antibiotic injection. The tap-and-inject technique was performed in the operating room using a blepharostat, following international protocol standards [8, 10]. The procedure included the administration of 5% iodine-povidone drops, collection of vitreous from different sites, and the intravitreal injection of a total of 0.05 ml of vancomycin 1 mg and 0.05 ml of ceftazidime 2.25 mg.

All patients received treatment within 48 h of experiencing their first symptoms. The approach was based on the availability of hospital resources at the time of infection. According to the records, each patient underwent a single procedure during the follow-up.

### Follow-up and OCT assessment

During follow-up, a complete ophthalmologic examination and Heidelberg macular OCT (Heidelberg Engineering, Heidelberg, Germany) imaging were performed. The OCT findings from the 1-year evaluation were included in the statistical analysis.

### Statistical analysis

Descriptive analyses were performed, and the means, standard deviations, and 95% confidence intervals (CIs) were calculated for continuous variables. For statistical analysis, the patients who were qualitatively reported to have counting fingers, hand motions, LP, or no LP vision were assigned logarithm of the minimum angle of resolution (logMAR) VAs of 1.80, 2.30, 2.80, and 3.00, respectively.

To analyze and compare the outcomes of the different treatment approaches, one-way analysis of variance and the Kruskal-Wallis test for continuous variables were performed with an alpha level of 0.05; the non-parametric Mann-Whitney test was used when only two groups were compared with an alpha level of 0.05. The OCT changes were analyzed using Fisher’s t-test and multivariate logistic regression. The data were collected using Excel version 16 software (Microsoft, Redmond, WA, USA) and exported to IBM SPSS Statistics for Windows (IBM Corp., version 25.0, Armonk, NY, USA) for statistical analysis.

For the statistical analysis among the groups, we only used data from the 23 positive vitreous culture patients, in order to exclude the bias of noninfectious cases.

## Results

Between January 2011 and December 2019, a total of 12,441 intravitreal anti-VEGF injections were administered. Out of these injections, 25 eyes of 25 patients were included in the study due to the development of acute endophthalmitis following the injections.

The calculated incidence rate of endophthalmitis was 0.20%, which translates to one case occurring for every 498 intravitreal injections performed.

Table 1 displays the distribution of injections and the corresponding incidence rates of endophthalmitis for each year. Notably, the years 2014 and 2017 had the highest infection rates, with 9 out of 25 cases (36.0%) and 6 out of 25 cases (24.0%), respectively.

**Table 1:** Injections and Endophthalmitis Events distributed between 2011 and 2019

	2011	2012	2013	2014	2015	2016	2017	2018	2019	TOTAL
Number of Injections	319	459	154	1759	1123	1376	1912	2017	1972	**12,441**
Endophthalmitis Suspicion	1	1	2	9	2	2	6	0	2	**25**
Positive Culture	0	1	2	8	2	2	6	0	2	**23**
Positivity (%)	0%	100%	100%	88,9%	100%	100%	100%	0%	100%	**92,0%**
Incidence (%)	0,000%	0,218%	0,133%	**0,455%**	0,178%	0,145%	**0,314%**	0,000%	0,101%	**0,185%**

*The Years of 2014 and 2017 are intentionally highlighted*

These findings highlight the variation in infection rates across different years of the study period.

All patients in the study experienced clinical symptoms of acute endophthalmitis between 2 and 7 days (3.76 ± 2.00 days) after receiving intravitreal anti-VEGF injections. These symptoms included ocular hyperemia, pain, as well as inflammation in the anterior chamber and vitreous. These clinical signs were consistent with the diagnosis of acute endophthalmitis and were observed in all cases included in the study [3].

Vitreous cultures were positive in 23 out of 25 eyes included in the study, resulting in a positive culture rate of 92.0%. The most commonly isolated organisms from the positive cultures were *Staphylococci*, accounting for 82.6% of the cases. *Streptococci* were isolated in 13.0% of the cases. Additionally, there was one case where a rare organism, *Brevibacillus* spp., was identified. Table 2 provides further details on the distribution of these isolated organisms.

**Table 2:** Patient Demographic Data

	Worse Vision PPV (N = 8)	Injection (N = 5)	Better Vision PPV (N = 10)	Total (N = 23)	*P*
**Sex, N (%)**					0.145
Female	6 (75.0)	4 (80.0)	3 (30.0)	13 (56.5)	
Male	2 (25.0)	1 (20.0)	7 (70.0)	10 (43.5)	
**Age (years)**					0.238^†^
Mean ± SD	55.1 ± 20.1	54.2 ± 14.5	66.4 ± 12.0	59.8 ± 16.1	
Median (range)	60.5 (23.0 to 80.0)	60.0 (35.0 to 69.0)	70.0 (45.0 to 81.0)	67.0 (23.0 to 81.0)	
**Eye, N (%)**					0.856
OD	4 (50.0)	3 (60.0)	7 (70.0)	14 (60.9)	
OS	4 (50.0)	2 (40.0)	3 (30.0)	9 (39.1)	
**Indication IVI. N (%)**					0.685
AMD	3 (37.5)	1 (20.0)	5 (50.0)	9 (39.1)	
Angioid Streaks	0 (0.0)	1 (20.0)	0 (0.0)	1 (4.3)	
CRVO	1 (12.5)	1 (20.0)	2 (20.0)	4 (17.4)	
CSR	1 (12.5)	0 (0.0)	0 (0.0)	1 (4.3)	
DME	1 (12.5)	2 (40.0)	3 (30.0)	6 (26.1)	
PDR	1 (12.5)	0 (0.0)	0 (0.0)	1 (4.3)	
Preoperative	1 (12.5)	0 (0.0)	0 (0.0)	1 (4.3)	
**Pathogen N (%)**					0.729
*Brevibacillus spp.*	1 (12.5)	0 (0.0)	0 (0.0)	1 (4.3)	
*Staphylococcus spp.*	6 (75.0)	5 (100.0)	8 (80.0)	19 (82.6)	
*Streptococcus spp.*	1 (12.5)	0 (0.0)	2 (20.0)	3 (13.0)	

SD, standard deviation; IVI, intravitreal injection; AMD, age-related macular degeneration; CRVO, central retinal vein occlusion; CSR, central serous retinopathy; DME, diabetic macular edema; PDR, proliferative diabetic retinopathy; EVS, Endophthalmitis Vitrectomy Study

† Analysis of variance

Kolmogorov-Smirnov normality test for age (*P* = 0.627)

The patient demographic data are shown in Table 2. The mean patient age was 59.8 ± 16.1 years (23–81 years), and 13 (56.5%) were women. Among the patients with endophthalmitis, the indications for intravitreal injections were neovascular AMD (39.1%), DME (26.1%), central retinal vein occlusion (17.4%), and angioid streaks with neovascularization, central serous retinopathy with choroidal neovascularization, and preoperative injection (each 4.3%).

The logarithm of the minimum angle of resolution (logMAR) BCVA before infection was measured to be 1.15 ± 0.74. At the time of the initial endophthalmitis diagnosis, immediately before treatment, the logMAR BCVA was recorded as 2.14 ± 0.75. After one year of treatment, the logMAR BCVA improved to 1.56 ± 0.93 (Table 3).

**Table 3:** Summary of visual acuity stratified by time of infection and type of treatment

	Worse Vision PPV (N = 8)	Injection (N = 5)	Better Vision PPV (N = 10)	Total (N = 23)	*P*
**VA before infection - logMAR** ^**‡**^					0.395
Mean ± SD	1.43 ± 0.96	1.26 ± 0.83	0.92 ± 0.53	1.15 ± 0.74	
Median (Range)	1.75 (0.20 to 2.30)	1.30 (0.30 to 2.30)	0.90 (0.20 to 2.00)	1.00 (0.20 to 2.30)	
**VA right before treatment - logMAR** ^**‡**^					0.002*
Mean ± SD	2.80 ± 0.00	2.12 ± 0.30	1.79 ± 0.21	2.14 ± 0.75	
Median (Range)	2.80 (2.80 to 2.80)	2.00 (1.50 to 2.80)	2.15 (0.50 to 2.30)	2.30 (0.50 to 2.80)	
**VA after 1 year - logMAR** ^**‡**^					0.001*
Mean ± SD	2.42 ± 0.30	1.56 ± 0.39	0.96 ± 0.14	1.56 ± 0.93	
Median (Range)	2.90 (0.90 to 3.00)	1.50 (0.80 to 3.00)	0.90 (0.50 to 2.00)	1.00 (0.50 to 3.00)	

logMAR, logarithm of the minimum angle of resolution; SD, standard deviation;

*Statisticatlly significant difference

**Fig. 1:**
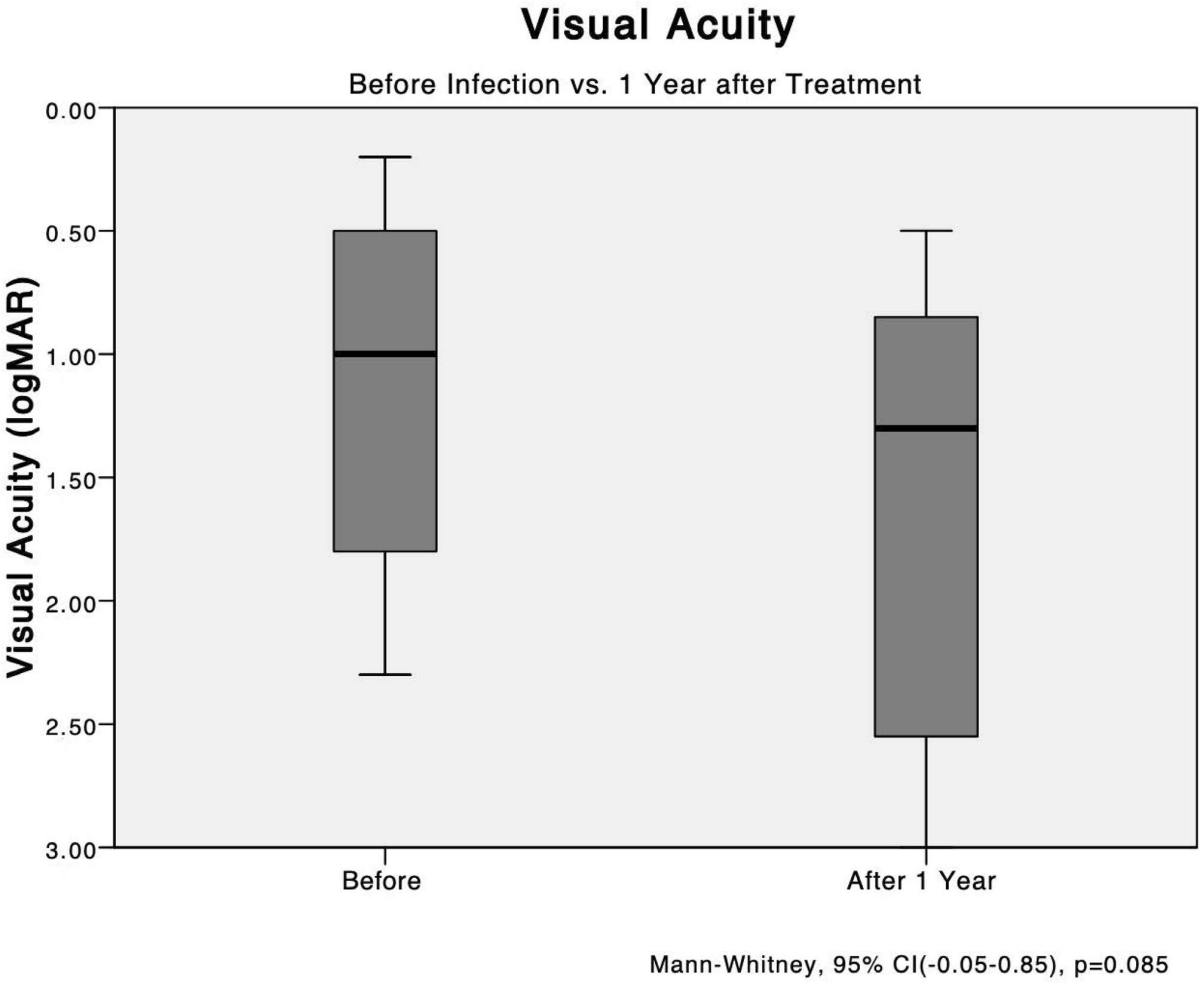
The mean VA levels before treatment compared with after 1 year. Analysis of all patients shows no significant difference between the logarithm of the logMAR BCVAs before and 1 year after development of endophthalmitis

Upon analyzing the data from all 23 patients, there was a marginal significance observed when comparing the BCVAs before and one year after treatment (P = 0.085). However, this difference did not reach statistical significance. Figure 1 illustrates this trend in visual acuity outcomes over the course of treatment. (Fig. 1)

Among the endophthalmitis cases, the injection group consisted of five patients (21.7%) who were treated solely with intravitreal vancomycin and ceftazidime. On the other hand, the PPV group comprised 18 patients (78.3%) who underwent 23-gauge PPV followed by the administration of IVAIES.

Within the PPV group, eight patients (34.8%) belonged to the worse-vision PPV subgroup, as they had a BCVA of LP immediately before the surgery. The remaining ten patients (43.4%) were categorized under the better-vision PPV subgroup, as they had a BCVA better than LP prior to the intervention.

**Fig. 2:**
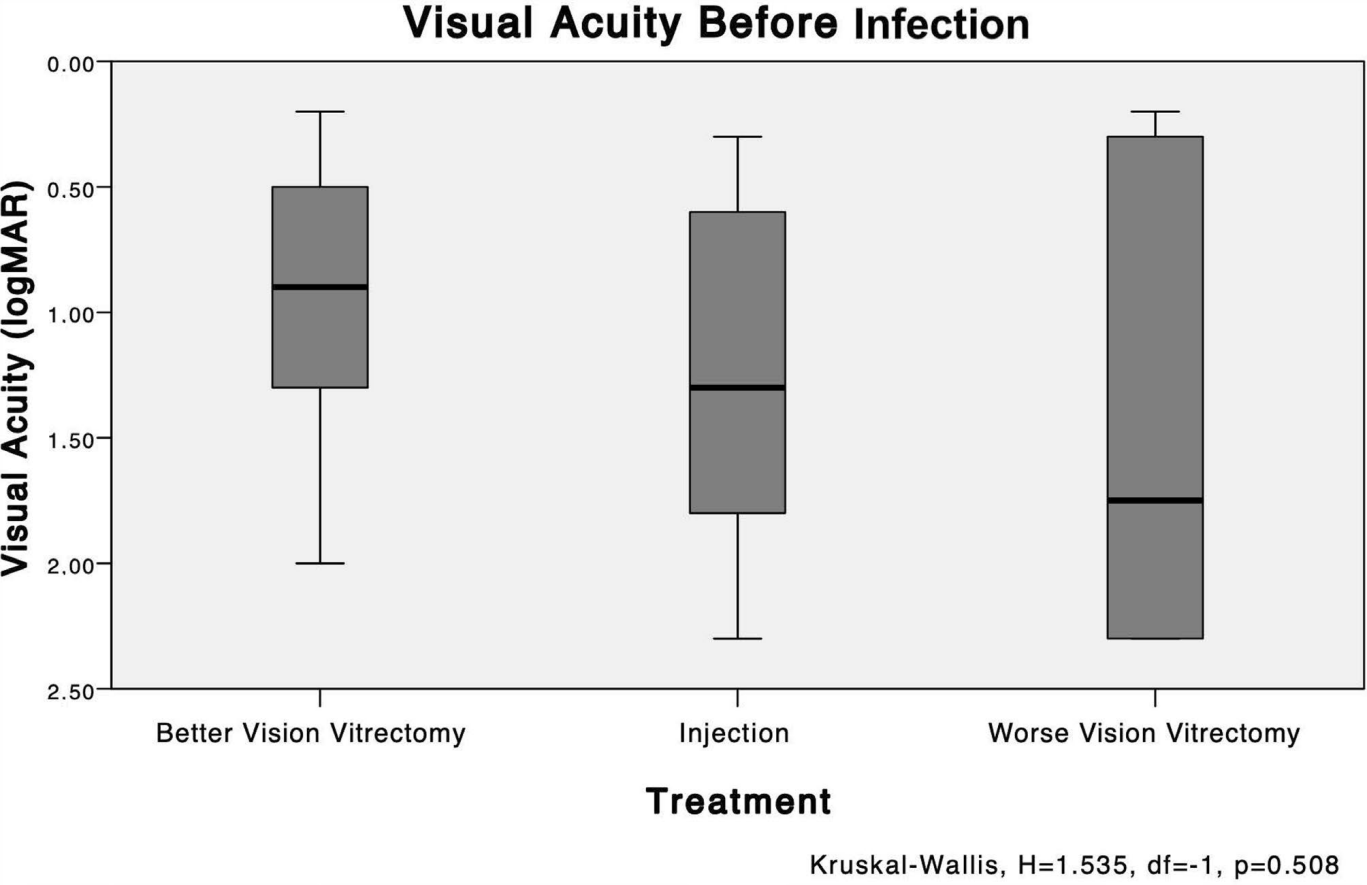
The mean VA levels before treatment: groups worse-vision PPV vs. injection vs. better-vision PPV. No significant difference in the logarithm of the logMAR best-corrected VAs is seen before the endophthalmitis developed (worse-vision PPV vs. injection vs. better-vision PPV, *P* = 0.508)

No significant differences in the BCVAs were seen before the endophthalmitis developed among all groups (worse-vison PPV vs. better-vision PPV, *P* = 0.301; injection vs. worse-vision PPV vs. better-vison PPV, *P* = 0.508) (Fig. 2).

Furthermore, the only significant difference in the BCVA during endophthalmitis (immediately before treatment) was seen between the two PPV groups (worse-vision PPV vs. better-vision PPV, *P* = 0.001; injection vs. worse-vision PPV, *P* = 0.101; injection vs. better-vision PPV, *P* = 0.357) (Table 3).

**Fig. 3:**
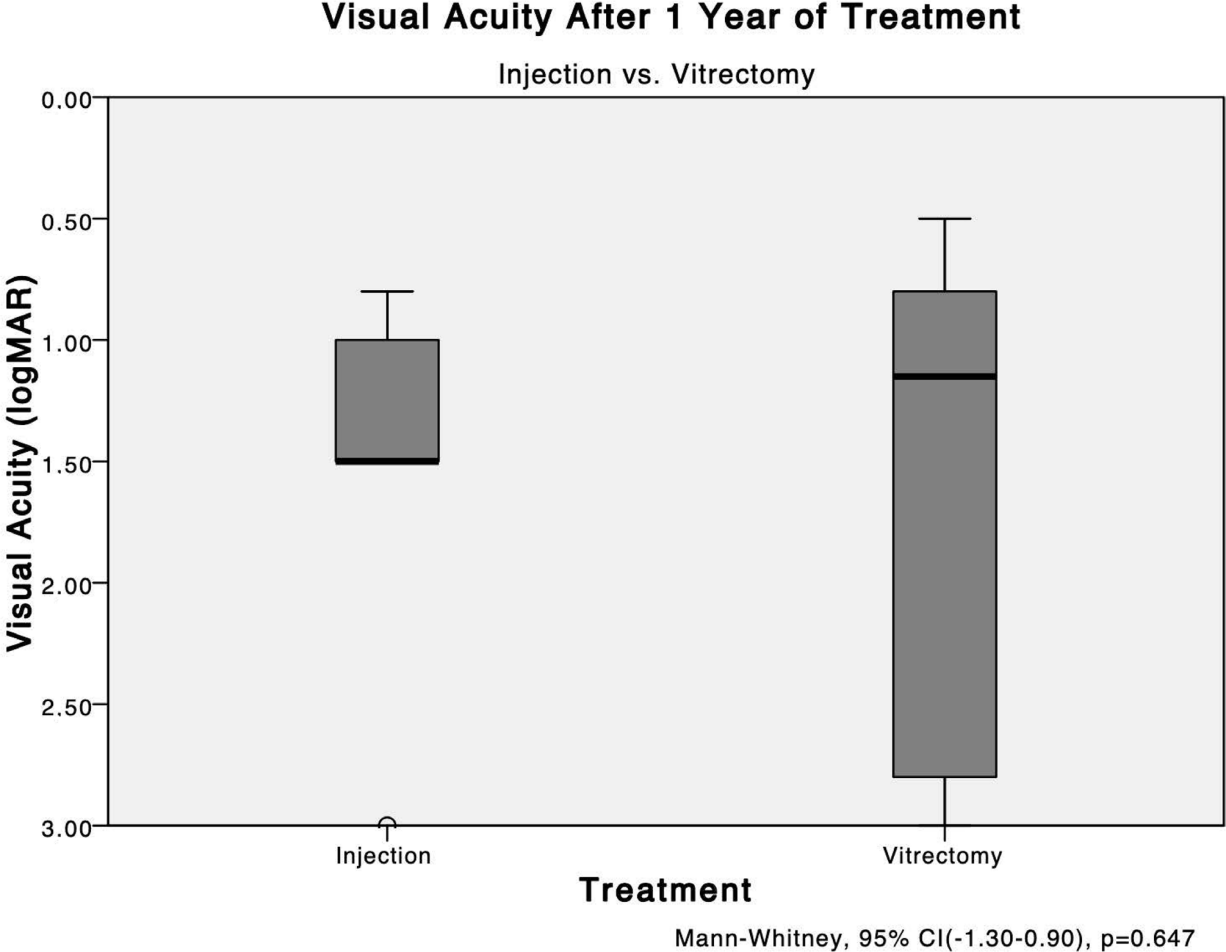
The mean VA levels after 1 year: injection vs. vitrectomy. The mean logMAR best-corrected VAs after 1 year of treatment in the injection group are 1.56 ± 0.86 and 1.60 ± 0.95 in the vitrectomy group. No significant (*P* = 0.647) difference is seen between them

The mean logMAR BCVA after 1 year of treatment in the injection group was 1.56 ± 0.86 and 1.60 ± 0.95 in the PPV group, a non-significant difference (*P* = 0.647) (Fig. 3).

A significant difference was observed between the worse-vision PPV group and the better-vision PPV group in terms of BCVA after 1 year of treatment. The better-vision PPV group exhibited better BCVA outcomes (0.96 ± 0.43) compared to the worse-vision PPV group (2.50 ± 0.83) with a significant difference (P = 0.005) according to the Mann-Whitney analysis.

**Fig. 4:**
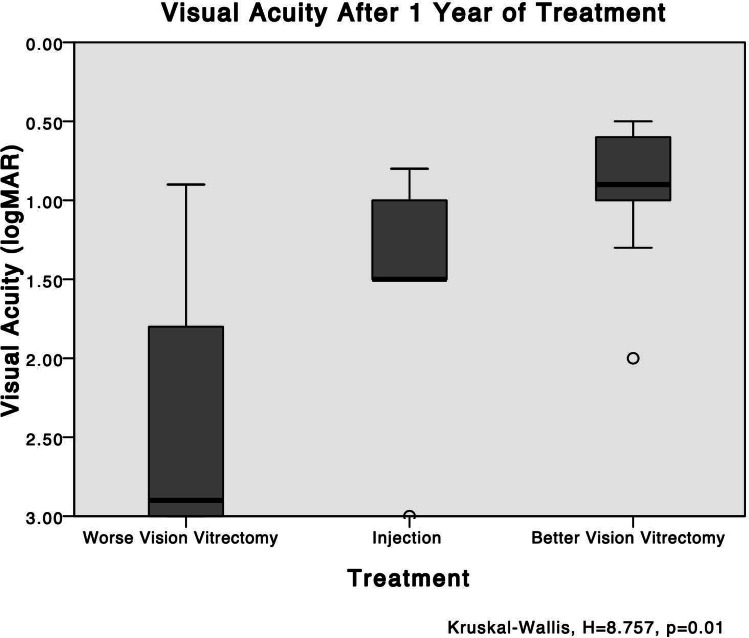
The VA levels after 1 year: worse-vision PPV vs. injection vs. better-vision PPV. A post-hoc analysis among the three groups shows a significant difference only between the vitrectomy groups, with better best-corrected VA after 1 year in the better-vision PPV group (*P* = 0.01)

Furthermore, a post-hoc analysis among the three groups (better-vision PPV, injection, and worse-vision PPV) also revealed a significant difference, specifically between the vitrectomy groups. The better-vision PPV group had significantly better BCVA after 1 year compared to the worse-vision PPV group (P = 0.01) when applying the Bonferroni correction. For a visual representation of the BCVA data and the differences between the groups, refer to Fig. 4. (Fig. 4)

The multivariate regression analysis revealed a significant difference in the final BCVA between the worse-vision PPV group and the better-vision PPV group. Patients in the worse-vision PPV group had a mean final BCVA of hand motions, while those in the better-vision PPV group had a mean final BCVA of 20/200. The difference in BCVA between these two groups was 1.42 logMAR unit (coefficient, 1.42; 95% CI, 0.71–2.12; P < 0.001), indicating that patients in the better-vision PPV group achieved significantly better visual outcomes compared to those in the worse-vision PPV group. This information is summarized in Table 4.

**Table 4:** Visual Acuity After 1 Year: Univariate and Multivariate Linear Regression

	Univariate Regression			Multivariate Regression	
	Coefficient (95% CI)	*P* Value		Coefficient (95% CI)	*P* Value
**Treatment**					
Better Vision PPV	Reference	-		Reference	-
Worse Vision PPV	1.45 (0.80 to 2.10)	< 0.001*		1.42 (0.71 to 2.12)	< 0.001*
Injection	0.60 (-0.15 to 1.35)	0.117		0.52 (-0.21 to 1.25)	0.164
**Sex**					
Female	Reference	-		-	-
Male	-0.61 (-1.35 to 0.13)	0.106			
**Age**	-0.02 (-0.05 to 0.01)	0.088		-	-
**IVI Indication**					
AMD	Reference				
DME	0.23 (-0.72 to 1.19)	0.632		-	-
CRVO	0.58 (-0.51 to 1.67)	0.294			
Other	0.96 (-0.13 to 2.05)	0.085			

CI, confidence interval; EVS, Endophthalmitis Vitrectomy Study; IVI, intravitreal injection; AMD, age-related macular degeneration; DME, diabetic macular edema, CRVO, central retinal vein occlusion.

*Statistically significant difference

The multivariate logistic regression analysis revealed a significant difference in the development of epiretinal membrane (ERM) between the worse-vision PPV group and the better-vision PPV group after 1 year of treatment. Patients in the worse-vision PPV group had a significantly lower likelihood of developing ERM compared to those in the better-vision PPV group (OR, 0.07; 95% CI, 0.01–0.82; P = 0.035). This suggests that the worse-vision PPV group had a reduced risk of ERM formation during the follow-up period. (Table 5)

**Table 5:** ERM Development after 1 Year: Univariate and Multivariate Logistic Regression

	Univariate Regression			Multivariate Regression	
	Odds Ratio (95% CI)	*P* Value		Odds Ratio (95% CI)	*P* Value
**Treatment**					
Better Vision PPV	Reference	-		Reference	-
Worse Vision PPV	0.04 (0.01 to 0.48)	0.012*		0.07 (0.01 to 0.82)	0.035*
Injection	0.17 (0.02 to 1.78)	0.138		0.38 (0.04 to 3.89)	0.419
**Sex**					
Female	Reference	-		-	-
Male	5.25 (0.87 to 31.55)	0.070			
**Age**	1.08 (1.01 to 1.17)	0.038		1.07 (0.98 to 1.16)	0.128
**IVI Indication**					
AMD	Reference				
DME	0.18 (0.02 to 1.52)	0.117		-	-
CRVO	0.33 (0.04 to 3.20)	0.341			
Other	0.04 (0.01 to 1.01)	0.057			

CI, confidence interval; EVS, Endophthalmitis Vitrectomy Study; IVI, intravitreal injection; AMD, age-related macular degeneration; DME, diabetic macular edema, CRVO, central retinal vein occlusion.

*Statistically significant difference

Table 6 displays the VA of each patient before endophthalmitis, immediately after treatment, and after 1 year of treatment. The table provides a comprehensive overview of the VA outcomes for individual patients throughout the study period.

**Table 6:** Visual Acuity Follow-up

	Injection Group					Better Vision PPV Group										Worse Vision PPV Group							
*Patient*	**1**	**2**	**3**	**4**	**5**	**6**	**7**	**8**	**9**	**10**	**11**	**12**	**13**	**14**	**15**	**16**	**17**	**18**	**19**	**20**	**21**	**22**	**23**
VA before infection	20/400	20/80	20/400	20/40	HM	20/63	20/50	CF	20/100	20/200	20/100	CF	20/400	20/30	20/200	20/32	No data	20/40	CF	HM	CF	No data	HM
VA right before treatment	CF	CF	CF	**LP**	**LP**	20/63	20/160	CF	CF	CF	HM	HM	HM	HM	HM	**LP**	**LP**	**LP**	**LP**	**LP**	**LP**	**LP**	**LP**
VA after 1 year of treatment	20/80	20/200	CF	20/100	**NLP**	20/100	CF	CF	20/70	20/200	20/200	20/200	20/400	20/100	20/80	**LP**	20/400	HM	20/125	**NLP**	**NLP**	**NLP**	**NLP**

*CF, Counting Fingers; HM, Hand Motion; LP, Light Perception; NLP, No Light Perception*

*LP and NLP are intentionally highlighted*

## Discussion

Endophthalmitis is the most feared complication of ophthalmologic procedures, and this concern also includes anti-VEGF injections. Similar to cataract extraction, endophthalmitis after anti-VEGF injections is associated with a poor visual prognosis despite prompt diagnosis [10].

The EVS [3] is the only randomized multicentric study that compared distinct treatment options for acute infectious endophthalmitis and proposed that PPV be performed in cases with an initial VA of LP [3, 7]. However, EVS is almost 3 decades old and included only patients who underwent cataract extractions. In addition, vitreoretinal surgery techniques have changed over the years, and improved retinal equipment has provided smaller transconjunctival trocars, higher cut vitrectomy rates, and a more stable surgery [9], with further predictable results, lower surgical risk, and better outcomes [11].

Since the publication of the EVS in 1995, there have been significant changes in ophthalmic practice patterns. Anti-VEGF intravitreal injections have emerged as the most commonly performed procedures in ophthalmology, representing a breakthrough in the treatment of macular diseases. As a result, millions of these injections are now administered annually, leading to an increased risk of acute endophthalmitis [12–14].

In cases of acute endophthalmitis, early PPV offers several advantages. It allows for the timely collection of vitreous material, facilitates the removal of inflammatory debris, and may enhance the effectiveness of antibiotics by providing a clearer vitreous cavity. Additionally, early surgery provides improved visualization, which helps prevent iatrogenic breaks and allows for the removal of denser vitreous opacities [15, 16].

In a meta-analysis conducted by Bande et al., the reported rates of endophthalmitis following intravitreal injections ranged from 0.012 to 0.10% [17], The most frequently isolated pathogens were coagulase-negative staphylococcus (38-65%) and Streptococci spp. (29-31%) [18]. The current study’s findings demonstrated a higher incidence of endophthalmitis compared to previously reported studies with similar pathogens. However, the differences in population characteristics, the use of anti-VEGF drugs, and the academic setting of our service may have contributed to these variations in incidence rates. Further research is necessary to determine the exact incidence of post-anti-VEGF endophthalmitis, particularly in low- and middle-income countries.

The increased incidence of endophthalmitis observed during 2014 and 2017 raises several potential hypotheses. Firstly, the use of repackaged bevacizumab syringes in 2014 may have contributed to the higher rates, as similar cases were reported by Edison et al. in 2013 [19]. Secondly, inconsistencies on the refrigerator system storage possibly may have led to increased contamination of the bevacizumab vials during the pooling method, as reported by Saoji et al. Thirdly, contact between the needle and the eyelashes or lid margins during the procedure may have played a role [8]. Lastly, in-training fellows may influence the elevated endophthalmitis incidence. These factors highlight the importance of maintaining strict protocols for medication handling, storage, and procedural technique to minimize the risk of endophthalmitis.

In our analysis, the time from initial symptom onset to diagnosis ranged from 2 to 7 days, which is consistent with the literature on acute endophthalmitis [15]. We observed positive culture results in 23 out of 25 cases (92.0%), which is a higher rate compared to previous reports [8, 20]. It is worth noting that the vitreous biopsy in our study was performed using a 23-gauge needle, while other studies have used 25-, 27-, or even 30-gauge needles. Interestingly, some reports have found no significant effect of needle gauge on the culture rate [12]. However, we routinely advocate for the use of a 23-gauge needle for vitreous sample collection, as it may allow for a larger volume of material to be obtained in the syringe, potentially explaining the higher positivity rate observed in our study.

Another possible explanation for the high positivity could be that patients receiving repeated anti-VEGF intravitreal injections might have more syneretic vitreous, which could facilitate the aspiration of a larger sample during the diagnostic procedure. Indeed, this topic could be a potential area for future studies. Further research could delve into the relationship between the type of intravitreal injection and vitreous characteristics, and how it might influence the occurrence and severity of endophthalmitis. Investigating this aspect could lead to valuable insights and advancements in the field.

In our study, the most common pathogen identified in culture samples was coagulase-negative *Staphylococci*, which accounted for 82.6% (19 out of 23) of the cases. This finding is consistent with previous reports [8, 21]. *Streptococci spp* species were the second most prevalent, identified in 13.0% (3 out of 23) of the cases. However, logistic regression analysis did not reveal any significant differences in the final BCVA among the different bacterial species (P = 0.097).

In our study, there were no significant differences in BCVA before infection among the groups. The BCVAs did not differ significantly between the injection group and the worse-vision PPV group (P = 0.921), the injection group and the better-vision PPV group (P = 0.684), or the worse-vision PPV group and the better-vision PPV group (P = 0.390). This allowed us to focus our analysis primarily on the BCVA after 1 year, indicating that the improved outcomes observed in eyes treated with PPV were not solely attributed to better preoperative prognoses.

The comparison between the BCVAs before and after 1 year among all patients revealed a marginally significant difference (P = 0.065), which may be attributed to the small sample size in our study. Additionally, it is important to note that the majority of BCVAs after 1 year were worse than the BCVAs before the development of endophthalmitis. This highlights the severity of the condition and the challenges in achieving optimal visual outcomes despite treatment efforts.

The comparison of BCVAs after 1 year between the two vitrectomy groups (better-vision PPV vs. worse-vision PPV) revealed a significant difference, favoring the surgical approach performed with a BCVA better than LP (P = 0.003). Furthermore, Dunn’s post-hoc analysis among the three groups demonstrated that the worse-vision PPV group experienced a significantly worse decrease in BCVA after 1 year (P = 0.01). Moreover, the multivariate regression analysis indicated a 1.42-logMAR difference in the final BCVA, favoring the better-vision PPV group. These findings suggest that better initial BCVA results in improved visual outcomes if vitrectomy is performed early.

Despite the better results in the final BCVA, epiretinal membranes developed more frequently in the better-vision PPV group after 1 year of follow-up. We propose that this finding could be explained by the surgical intervention, since the posterior vitreous detachment could lead to hyalocytes deposition on the retinal surface and stimulate ERM formation [22–24].

The findings of this study support the notion that worse initial visual acuity (LP or worse) during endophthalmitis presentation is associated with poorer visual outcomes. In addition, the observation that 50% of eyes with LP immediately after treatment deteriorated to no light perception (NLP) after 1 year suggests that the severity of the initial endophthalmitis may influence the long-term visual prognosis. These findings raise questions about the treatment protocol advocated by the EVS, which recommended a surgical approach only for cases with LP or worse visual acuity. Over time, numerous studies have challenged the EVS protocol and proposed alternative approaches for managing acute endophthalmitis cases. The increasing use of early PPV has gained recognition and appears to offer potential benefits [13, 14, 16, 25].

Our findings corroborate those of Far et al., who conducted a meta-analysis that found significantly worse visual acuity outcomes after cases of endophthalmitis treated with intravitreal injections compared to PPV after cataract extractions [21]. These findings cast doubt on the generalizability of the EVS treatment protocol.

To the best of our knowledge, this current retrospective study is the first to describe and compare treatment approaches for endophthalmitis after intravitreal injections and suggest that pars plana vitrectomy (PPV) could be an effective treatment option. We observed significant results favoring a surgical approach, particularly in eyes with better initial visual acuity. This study also highlights concerns regarding the repackaging and pooling methods of bevacizumab, which may be associated with a higher risk of endophthalmitis development. Additionally, it raises questions about the role of early PPV in the management of acute endophthalmitis after cataract extraction, as recently discussed on a randomized clinical trial conducted by Sen et al. [26].

In contrast to the findings reported by Singh et al. [20], we believe that the higher rates of endophthalmitis observed in our study could be attributed to the pooling technique. Therefore, we propose that Bevacizumab should be divided into aliquots and packaged in single-use syringes by a pharmacy expert within a clean room environment, such as a laminar hood. These syringes should then be properly sealed and stored in a refrigerator maintained at a temperature of 2–8ºC, with regular biological control checks conducted on a weekly basis. By implementing these measures, we aim to minimize the risk of contamination and subsequent endophthalmitis associated with the use of Bevacizumab. Finkelstein et al. showed that the use of prefilled syringes significantly reduces this risk [27].

The current study possesses several strengths. Firstly, it is the first analysis to include a large number of Brazilian patients and incorporates previous infection ophthalmologic data, providing valuable insights into the local context. Additionally, the study compares and analyzes ancillary examination results, allowing for a comprehensive assessment of the topic.

However, there are certain limitations that should be acknowledged. Firstly, the study’s retrospective nature introduces inherent limitations such as potential selection bias and reliance on existing data. Furthermore, the data is derived from a single center, which may limit the generalizability of the findings to a broader population. Distinct surgeons proficiency may create influence the surgeries outcomes. Lastly, the relatively small number of endophthalmitis cases included in the study may impact the statistical power and precision of the results. We aim to enhance the future analysis by increasing the sample size through the inclusion of more multicenter data.

## Conclusion

In conclusion, the findings of this study suggest that PPV could be considered as a treatment option for patients with acute endophthalmitis following intravitreal injections, particularly in cases where the initial BCVA is better than LP. The use of advanced surgical techniques and equipment may contribute to improved outcomes in these cases. However, it is important to note that further research is required to validate these findings. Specifically, randomized controlled prospective studies or causal inference studies would provide stronger evidence to support the use of PPV in this context.

## Data Availability

The datasets used and/or analyzed during the current study are available from the corresponding author on reasonable request.
